# ‘It’s not because we don’t believe in it...’: Headteachers’ perceptions of implementing physically active lessons in school

**DOI:** 10.1186/s12889-019-8021-5

**Published:** 2019-12-12

**Authors:** Ingrid Skage, Sindre M. Dyrstad

**Affiliations:** 1 Department of Education and Sport Science, University of Stavanger, 4036 Stavanger, Norway; 2 Department of children, youth and education, Municipality of Stavanger, 4068 Stavanger, Norway; 3 Department of Public Health, University of Stavanger, 4036 Stavanger, Norway

**Keywords:** School-based, Physically active lessons, Dissemination, Headteachers, Qualitative

## Abstract

**Introduction:**

Implementation of school-based physical activity (PA) programmes has proven to be difficult, particularly due to schools’ focus on academic performance and lack of organisational support for PA interventions. However, physically active lessons (PA integrated into academic lessons) holds promise as a teaching method that increases children’s PA levels without reducing academic time. Headteachers play a significant role in facilitating change in school, but little is known about headteachers’ attitudes towards physically active lessons and their benefits. The purpose of this study was to explore headteachers’ perceptions of physically active lessons, and identify factors affecting headteachers’ acceptance or rejection of physically active lessons implementation.

**Method:**

A total of 29 semi-structured telephone interviews were conducted with headteachers in primary and secondary schools in the city of Stavanger, Norway. Adopting a phenomenological approach, qualitative data were analysed using inductive content analysis.

**Results:**

Although most of the headteachers believed that physically active lessons could contribute positively to pupils’ health and learning, only four of 29 schools decided to proceed with implementation. Physically active lessons were more likely to be adopted when the intervention addressed a clearly defined priority area at the school. Change overload and lack of in-depth knowledge of physically active lessons’ function and intent appeared to be the most important factors for choosing not to implement physically active lessons.

**Conclusion:**

One of the major challenges for headteachers was deciding which of the many proposed changes the school should prioritise. If physically active lessons was to be prioritised by headteachers it is very important to communicate thoroughly to the headteachers what the schools can achieve by implementing physically active lessons and how the innovation aligns with school policies and goals. Given the flexibility inherent in physically active lessons and the schools’ differing needs and priorities, it was important to emphasise to headteachers that physically active lessons could be adapted to different local school contexts.

## Introduction

In Norway, 9- and 15-year-olds engage in sedentary behavior between 7.5 and 9 h per day, respectively, and 46–72% do not reach the recommended 60 min of daily physical activity (PA) [1, 2]. Globally, it is recommended that all schools develop policies to address PA during the school day for increasing children and young people’s PA [3]. Despite a growing movement to develop and adopt PA interventions in school, adoption of school-based PA interventions has proven to be challenging. Schools tend to prioritise academic performance over health-related outcomes, and they often lack organisational support for PA interventions [4–6].

Physically active lessons are designed to increase children’s PA levels without reducing academic time by integrating PA into lessons in learning areas other than physical education (Watson et al., 2017). Recent systematic reviews and meta-analyses have related physically active lessons in school to improved health, enhanced cognitive function and increased academic performance [7–10].

The Active School Programme originated in the city of Stavanger, Norway in 2012, with the goal of increasing children’s PA level in school, in order to improve health and learning. The core intervention component was physically active lessons. After a successful pilot study in 2013–14 [11], a 10-month cluster randomised controlled trial in primary schools was conducted in 2014–15. It was found that increased PA in school tended to benefit children’s cognitive function, as well as increase aerobic fitness for the least fit children [12, 13]. Results from process evaluation showed that physically active lessons were highly appreciated by both teachers and children [14]. Similar findings have also been reported by others [15, 16].

Despite physically active lessons’ apparent effectiveness as a method to increase children’s daily PA, and its facilitative role regarding prioritised academic goals, little is known about the factors that affect school adoption of physically active lessons. Teachers are the most important agents for bringing change and innovation in educational practice [17], but the importance of the headteachers’ role in school improvement work, including choosing between different programmes, has also been acknowledged [18–20]. Given headteachers’ significant role, information about adoption factors such as headteachers’ attitudes towards physically active lessons and prioritisation, would be useful for developing targeted strategies for increasing physically active lessons adoption and implementation. Thus, the aim of this study was to explore headteachers’ perceptions of physically active lessons, and to identify factors affecting headteachers’ approval or rejection of physically active lessons implementation.

### Schools readiness for adoption

In the Norwegian context, increased PA for children and youth has been high on the political, educational and research agendas, and a recent report stated that schools are one of the dominant locations for sedentary behavior, especially due to sedentary traditional teaching in the classroom [2]. With this background, the Norwegian government recently added a goal of including one hour of daily physical activity for all children in school, without extending the school day or compromising teachers’ pedagogical autonomy [21]. This implies that schools have considerable autonomy in organisation of physical activity, and their priorities and ability to implement change are likely to impact adoption of physically active lessons.

Introducing new interventions in school is a complex and challenging process. According to the literature on planned change, the implementation process consists of three phases: initiation, implementation and institutionalisation [19, 20]. The initiation phase addresses schools’ initial considerations of whether they are ready to adopt an intervention [22]. According to Leithwood (2018), surprisingly little research has investigated headteachers’ response to external change initiatives. Spillane et al. [23] suggest that a headteacher’s response to an external change initiative is influenced by their existing knowledge, the vision they have for their own school and the beliefs and values they hold about what is important to them professionally. Previous research has shown that headteachers were more willing to adopt a physical activity intervention if it addressed educational outcomes in addition to health promotion [5, 24, 25]. Furthermore, Domitrovich et al. [18] noted that interventions that aligned directly with a school’s mission, priority areas and existing practise, are more likely to be prioritised.

While headteachers must answer to external expectations, they also rely on teachers’ motivation to perform the necessary work. Teachers are more likely to be committed to implementing an intervention if they have played an active role in the decision-making process and perceive that the intervention meets prioritised needs [19]. Previous research has shown that teachers were more likely to involve themselves in a school development activity when the headteacher played an active role [26]. Hall and Hord [17] have, through extensive empirical research of headteacher leadership, identified three distinct change facilitator styles. The *initiator* is always thinking ahead and makes decisions based on what they believe will benefit the pupils. The *manager* focuses on formal policies and protecting staff, and the *responder* lets others take the lead and tends to downplay the significance of proposed change.

## Methods

### Design and participants

The current study has a qualitative research design with an inductive approach where patterns and themes were identified from the data [27]. Data were collected through semi-structured telephone interviews. This interview type is an appropriate method that provides in-depth knowledge of headteachers’ perceptions and prioritisation regarding adoption and implementation of physically active lessons [28].

To expand the use of physically active lessons, in the autumn 2017, all 40 primary and secondary schools in the municipality of Stavanger, Norway were invited by the “Active School” project team to implement physically active lessons and contribute to the project’s website that provides free access to high quality lessons. After a short briefing meeting with all headteachers, where rationale for physically active lessons was presented, invitations for application were sent by e-mail from local school authorities, who encouraged their schools to participate. All participants received written information about the study and gave their written consent to participate in the interviews. The study was approved by the Norwegian Social Science Data Services.

### Interviews

Telephone interviews were conducted during the spring of 2018. Headteachers received an invitation to participate in telephone interviews through mail and follow-up reminders. Five headteachers from the schools that had participated in the effectiveness evaluation in 2014–15, and four new employees without knowledge of the request were excluded, and two headteachers did not respond. In total, 29 of 40 headteachers in primary and secondary schools in Stavanger participated. Sixty-two percent were female and 38% were male, and the mean age was 53 years (ranging from 39 to 70 years). Participating schools were all from urban areas, within an average distance of 7 km from the local university. A summary of headteachers` demographic is presented in Table 1.

**Table 1:** Demographics for the 29 headteachers interviewed in the study

Age (years)	Gender	Years of experience working as headteacher	Numbers of school-students	Type of school (grades)
47	F	11	487	1–10
44	M	6	330	8–10
70	F	24	407	1–7
61	F	6	524	1–7
48	M	6	349	1–7
69	F	23	366	8–10
58	F	6	308	1–7
48	M	4	287	8–10
58	M	8	432	1–7
64	M	10	650	1–7
60	F	8	386	1–7
46	M	2	408	8–10
59	F	3	485	1–7
56	F	9	368	1–7
59	F	10	240	6–10
48	F	3	372	1–10
63	M	8	395	1–7
41	M	4	310	8–10
45	M	4	117	1–7
55	F	6	455	1–7
52	F	7	207	8–10
48	F	10	345	8–10
42	F	2	372	1–7
57	F	3	321	8–10
39	M	2	328	8–10
65	F	10	341	1–7
46	F	7	307	1–7
57	F	12	658	1–7
43	F	4	70	1–7

*F* female; *M* male

A semi-structured interview guide with open-ended questions was developed on the basis of central components identified in the literature about introducing new practices in school [19, 20]. Interview questions were sent by mail ahead of the telephone interviews and an appointment was scheduled for each interview. The telephone interviews were scheduled for 15–20 min, and lasted between 7 and 24 min (average 16 min). The interviews requested data on perceptions, prioritisation and response to the request to introduce physically active lessons.

### Data analysis

Data from telephone interviews were recorded and transcribed in full. The transcripts were read and reread by the interviewer to ensure accuracy of the data. Data were analysed using a qualitative content analysis, focusing on the manifest content [27]. This approach focuses on subject and context, and is designed to describe similarities and differences in transcripts from e.g., interviews [29]. The analysis was an iterative process, with all data processed in the computer programme QSR NVIVO11. Initially, each interview transcript was analysed as a single case. All text from the interviews was divided into meaning units that were condensed to abstracting data from the full body of transcripts, and thereafter coded using an inductive approach. A short summary of each interview was written. Similarities and differences in headteachers’ responses were identified. Patterns were labelled and grouped into categories and subcategories. In the later stages, existing theoretical perspectives such as the Quality Implementation Framework [22], focusing on the first phase of implementation, addressing critical steps before the implementation begins (conducting a need, innovation-organisational fit and capacity assessment), were integrated to get a more complete understanding of the data.

To ensure trustworthiness of the coding and interpretation of the data initially, findings were discussed among authors as recommended by Kvale et al. [30]. Quotations from interviews are used to illustrate the findings. To safeguard confidentiality as much as possible, some information is omitted. Only the position as headteacher is given. The interviews were conducted in Norwegian and selected quotations were subsequently translated to English.

## Results

In total, four of 29 schools decided to proceed with implementation of physically active lessons. Headteachers’ perceptions and prioritisation were assessed by initial considerations, as were their perceptions of need of the intervention, compatibility with plans and work, and capacity to implement. Table 2 summarises the themes, main categories, subcategories and positive/negative quotes identified for headteachers’ perceptions and prioritisation regarding implementation of physically active lessons.

**Table 2:** Sample quotes for themes, main categories, subcategories, and positive/negative quotes identified for Headteachers’ perceptions and prioritisation regarding implementation of physically active lessons

Themes	Main categories	Subcategories	Sample quotes	Positive (+) /negative (−) quotes for implementation
Perception of need	Benefits	Increased learning (n:18)	“I believe physically active lessons is a fine way to get engagement from the children who do not learn so much from just sitting quietly in the classroom and listening to the teacher.”	+
		Reduction of sedentary time (n:7)	“In the lower secondary school, the ordinary lessons are based on the pupils sitting still at their desks, even the breaks are not active since they are mostly socialising with others using digital gadgets … This makes me think that it is important that they do other activities, involving physical activity.”	+
	Identified needs	New knowledge (n:2)	“We wanted to participate in order to further develop our own model, we were afraid of missing out on new knowledge in the area.”	+
		For the six-year-olds (n:5)	“When the six-year-olds came into school, they should just play, and then it has become more and more teaching subjects and less and less time for play, and we have discussed this and agreed on that we really want to do something about it.”	+
Perception of compatibility	School policy	National curriculum (n:10)	“Physically active lessons are relevant to our school because we have worked a lot with understanding the national curriculum …. and it adds up to a lot of children activities.”	+
		Municipality quality plan (n:6)	“You know, last year at this time, we were in the process of implementing the new quality plan for the municipality, but it says nothing about physical activity but reading, writing, maths and digital competencies, so we thought it didn’t fit.”	-
	Local school priority areas	Healthy lifestyle (n:11)	“We are positive to physically active lessons because we have the focus area healthy lifestyle and invest a lot of time in both physical and mental health.”	+
		Variated teaching (n:12)	“We are focusing on introducing varied and more practically supported teaching and it is positive to bring in more physical activity, and the teachers are always looking for good tools they can use in teaching.”	+
		Have facilitated physical activity (n:12)	“I see many links between what you presented in Active School programme and what’s called Active TL-programme (a pupil-driven commercial PA program) So my thinking was, here we are reasonably well covered.”	–
		Better suited for primary school children (n:2)	“We want more physical activity in school but jumping the multiplication table and play-based activities, it seems a bit too childish in the secondary school.”	–
Perception of school capacity	Change experience	Culture for change (n:14)	“Fifteen years ago, this school was very traditional, but we have worked systematically with development work, and now we perceive ourselves as a learning organisation”	+
		Struggling with change work (n:12)	“The teachers work the way they always have done, therefore it is challenging to initiate change work, and teachers are rarely excited about new proposals. But I believe it is not typical for us, it is the way it is.”	–
	Change overload	Competing priorities (n:24)	“As school, we get so many offers on important things we can participate in, and as headteacher, you have to prioritise. The reason why we did not go for it (physically active lessons) wasn’t because we don’t believe in it, but because the teachers have enough and we don’t have capacity for everything new.”	–
		Teachers are tired of change (n:14)	“An employee survey we recently conducted showed that the teachers are tired of change.”	–
	Leadership	Capacity to support implementation (n:11)	“Should we have implemented physically active lessons, then it must be done collectively, but in this case neither I nor the management have had capacity to do the necessary work.”	–
		Management decides (n:16)	“I was the one who decided not to participate in the project; I discussed it with the management team, but not the teachers.”	–
		Collective decision (n:13)	We made the decision not to participate together with the teachers, and everyone agreed that the time was not appropriate. Physical activity is an area I’m passionate about, so that decision was to compromise my own values.”	+
		Teachers’ autonomy (n:4)	“It is not appropriate for us to enter the project in the short term, but I know there are some teachers who use this as a didactic tool already.	–
	Incentive for change	Funding(n:3)	“An important contributing factor to our acceptance of the School in Motion project (national research project for increased PA in lower secondary school) was that it came with extra funds … I think that’s what it takes.”	–
		Stronger knowledge-base (n:3)	“I considered starting with physically active lessons, but the biggest challenge is to get the teachers exited, I think it would have been easier to get buy-in if there had been documented learning outcomes of the method.”	–

n: Refers to how many headteachers gave similar comments

### Perceptions of need

Headteachers believed that physically active lessons could contribute positively to pupils’ learning and health. Primary school headteachers felt that physically active lessons could address children’s need for a varied teaching approach. Secondary school headteachers felt that physically active lessons could address more general concern about adolescents’ physical and mental health, referring to observations of increased sedentary behaviour. In total, 25 teachers in four schools agreed to implement physically active lessons. Of the reasons headteachers mentioned for acceptance of physically active lessons, first and foremost was the possibility of adding to existing knowledge in a prioritised area. Additionally, headteachers accepted physically active lessons based on a decision made to introduce more PA and play-based learning for the youngest children.

### Perceptions of compatibility

The main view of the headteachers was that physically active lessons were consistent with the national curriculum. There were some different perceptions regarding whether physically active lessons were in line with the local school authority’s policy, due to the fact that physical activity was not explicitly described as a priority area in the municipal quality plan. However, most of the headteachers perceived physically active lessons as a useful tool and aligned the innovation to a variety of prioritised areas, including variation in teaching approach, practical supported teaching, relationship competence, and physical and mental health. Most of the headteachers perceived facilitating increased physical activity in school as an important task. However, when considering the need for adopting physically active lessons, a majority of the participants reported they already had sufficient activities to increase physical activity in school (e.g., access to sports halls, outdoor school, and physical activity during recess). Furthermore, two secondary school headteachers considered physically active lessons to be too childish for young people.

### Perception of school capacity

Despite the value placed on physically active lessons and the fact that most of the headteachers perceived the innovation to be suited to the school’s priority areas, it was obvious that challenges associated with school development and prioritisation between competing daily tasks were demanding. The participants frequently mentioned focus on national tests and academic achievement as limiting factors for participation in new projects. Furthermore, a few headteachers mentioned that lack of documented learning outcome and funding influenced their decision to reject physically active lessons. Headteachers’ capacity to support implementation was also a challenge. The majority of headteachers had not informed or discussed the possibility of adopting physically active lessons with the teachers in their school, and the decision not to participate was made by the headteacher either alone, or together with management. Although many headteachers talked about previous successful change work, they sensed that the teachers were generally weary of change, and that it was therefore challenging to motivate teachers to support new change initiatives. However, some headteachers reported that individual teachers had started using physically active lessons on their own initiative.

## Discussion

The aim of the study was to investigate headteachers’ perceptions of physically active lessons, and identify factors affecting acceptance or rejection of physically active lessons implementation. Even though most of the headteachers believed that physically active lessons could contribute positively to pupils’ health and learning, only four of 29 schools decided to proceed with implementation. Physically active lessons were more likely to be adopted in schools where the intervention met defined priority areas at the school. Change overload and lack of in-depth knowledge of physically active lessons’ function and intent appeared to be the most important factors for refusing to implement physically active lessons.

### Perception of need

Most of the headteachers had the perception that physically active lessons could contribute positively to pupils’ health and learning. According to Greenberg et al. [19], perception of benefits for the target audience is an important factor affecting a decision to adopt a programme. Furthermore, the results indicated that primary school headteachers acknowledged that pupils have different learning styles, and that PA is important for children’s wellbeing and motivation for learning. In support, a recent study found that addition of physically active lessons was associated with a significant increase in academic performance for low-performing children [31], and simultaneously benefitted all demographic subgroups [32]. However, secondary school headteachers considered physically active lessons more as a means of integrating PA for health into the school day, and less as a means of improving learning. Physically active lessons provide the means to achieve a dose of PA sufficient to improve health while also improving learning [7, 33]. However, the results of this study indicate that PA and learning, to some extent, are understood as two separate activities and not integrated into a single activity as intended by physically active lessons. This finding indicates lack of clarity about goals and means, which Fullan [20] emphasises is a persistent challenge in implementation processes.

### Perceptions of compatibility

Many headteachers perceived physically active lessons as compatible with the national curriculum. However, there were different interpretations of local school policy, which affected some of the headteachers’ priorities. The decision not to adopt physically active lessons may be an expression of a leadership style that emphasises the administrative aspect of leadership, which is committed to following the correct application of rules and policy and does not typically initiate attempts to move beyond the basis of what is required [17]. An interesting finding is that while most of the headteachers perceived facilitating PA as an important task in school, studies from other countries have reported the opposite [24, 25]. The fact that the majority of schools reported that they already had strategies for increased PA beyond physical education support this finding. Furthermore, many of the participants perceived physically active lessons to be in line with work already going on in the school. According to Domitrovich et al. [18], it is easier to implement new initiatives within existing practise. Nevertheless, when considering adopting physically active lessons, it looked like the majority of headteachers ticked off “we already do that in our school” on an imaginary list. However, most of the PA strategies they mentioned were not aligned to teaching and learning. According to Spillane et al. (2006, p. 50–51), people tend to give more credence to information that confirms rather than challenges or refutes their understanding. The results of this study indicated that the headteachers were not convinced that physically active lessons were necessary to implement, given that pupils had plenty of opportunities for PA during the school day. This finding indicates that the majority of headteachers lacked an in-depth understanding of physically active lessons’ function and intent.

The results indicated that physically active lessons are more likely to be adopted in schools where the innovation meets a clearly defined priority or improvement area at the school. Schools as implementing organisations are faced with overloaded improvement agendas. If physically active lessons is to be prioritised, it must not only be perceived as important, but also important relative to other needs [20, 22]. It is important that headteachers understand what the innovation consist of and what using it entails to be able to make an informed and well-thought-out decision about adoption of physically active lessons. But as Fullan (2016, p. 70) has pointed out: “people often don’t know what they don’t know”. As a consequence, school leaders need to be thoroughly informed about physically active lessons’ function and intent, and programme developers need to help schools understand how physically active lessons can be embedded in school policy and goals. Furthermore, it must be emphasised that physically active lessons are “a part of” achieving prioritised academic and educational goals, and not “an addition to” their workload.

### Perception of school capacity

The majority of headteachers had the perception that physically active lessons could contribute positively to pupils’ health and learning. However, the perception of benefits for the pupils was apparently not sufficient to trigger school engagement, since only four of twenty-nine schools (three primary and one secondary) actually adopted physically active lessons.

A common thread throughout the interviews was headteachers’ perceptions of change overload. The headteachers seemed to be influenced by both perceived pressure from other school development projects, and teachers’ lack of motivation to conduct new change initiatives. In support, previous research has shown that headteachers face many expectations, leading to tension with many dilemmas [34]. Although many headteachers describe previous positive change experience and climate for change, which indicates general capacity for change, [35], they generally considered it challenging to motivate teachers to adopt new change initiatives. The fact that the majority of headteachers did not inform the teachers about the request to participate in the “Active School” project, supports this finding. The decision not to inform the teachers may reflect a *manager leadership* style, which emphasises protecting staff and tending to need more knowledge and time to prepare for an efficient implementation [17]. Indeed, some headteachers called for stronger evidence for outcomes relevant for them. Faced with overloaded improvement agendas, this strategy may also serve to protect teachers from random change initiatives, thus contributing to balanced change and stability [36]. Starting multiple change projects simultaneously may result in too little time spent on each project and not enough time to carry out the learning process needed for successful implementation. Only a few headteachers mentioned funding as a motivating factor for choosing a specific programme. This finding indicates that funding is not crucial for acceptance, though it makes it easier to accept change initiatives.

Many headteachers experienced lack of capacity to lead change work. Some did report that teachers had started to teach physically active lessons of their own initiative, regardless of participation in the “Active School” project. This kind of leadership may reflect a *Responder* facilitator who tends to minimise the significance of proposed change and leave the pedagogical work to the teachers [17]. However, even though “innovation champions” are acknowledged as important for sustainable implementation [22], headteachers need to be involved and draw attention to the importance and relevance of the project [20, 26].

### Strengths and limitations

To our knowledge, this is the first study to examine headteachers’ perspectives on adoption and implementation of physically active lessons, and the results move beyond teacher and pupil views that dominate the current literature [33]. Another strength is a large number of interviews, resulting in achieving data saturation [28]. While the study outcomes are Norwegian-centric, results may be used to influence physically active lessons implementation in culturally similar countries. A limitation was that the participants were aware that the lead author who conducted the interviews was also a member of the “Active School” project team. This may have influenced them to respond more positively towards physically active lessons than they would otherwise have done. It should also be mentioned that data was obtained in a university city where there is a greater pressure to recruit schools for research projects than in more rural areas. That is, schools in Stavanger may be exposed to a great number of research requests, contributing to increased reporting of change overload.

## Conclusion

The majority of headteachers believed that physically active lessons could contribute positively to pupils’ health and learning. However, perceptions of benefits for the pupils were not sufficient since only four of 29 schools adopted physically active lessons. There were different opinions as to whether the innovation was in line with local school policies, and the results indicate that physically active lessons were more likely to be adopted in schools where the innovation met a clearly defined priority area at the school. Change overload and lack of in-depth knowledge of physically active lessons’ function and intent, appeared to be the most important factors for rejection of physically active lessons. Schools as implementing organisations have numerous of goals. To make headteachers better qualified to make decisions about adopting physically active lessons, it is very important to specify what schools can achieve by implementing physically active lessons, and how the innovation aligns with school policies and goals. Furthermore, physically active lessons facilitative role regarding achieving prioritised educational goals, and not solely increased teacher workload, must be emphasised. Given the flexibility inherent in physically active lessons, and schools’ different needs, it is important to emphasise that physically active lessons can be adapted to the individual schools’ improvement priorities. This study points to the usefulness of the Quality Implementation Framework in studying headteachers` perceptions regarding an implementation of physically active lessons. Consistent with this model, a planned introduction, focusing on knowledge relevant to school leaders and teachers and offering practical demonstration focused on adapting to context, may facilitate greater buy-in and implementation of physically active lessons.

## Data Availability

The dataset analysed during the current study are available from the corresponding author on reasonable request.

## Abbreviations

CBAM: Concern based adoption model
LoU: Level of use
PA: Physical activity
